# Extended Telemonitored Follow-Up After Acute Coronary Syndrome: A Healthcare Pathway That Improves Cardiovascular Prevention and Patient Experience, and Reduces Outpatient Visits †

**DOI:** 10.3390/jcm14207283

**Published:** 2025-10-15

**Authors:** Ernesto Dalli-Peydró, Alicia Serrano-Romero, Rocío Serrats-López, Alvaro Salvador Minaya-Zaballos, Alan Herrera-Vásquez, Sofía Ramírez-Candela, Angela Arias-Fresneda, Alejandra Llanos-Gabaroa, Nuria Muñoz-Ramos, Amparo Fresneda-Fresneda, Juan Cosín-Sales

**Affiliations:** 1 Servicio de Cardiología, Hospital Arnau de Vilanova, 46015 Valencia, Spain; dalli_ern@gva.es (E.D.-P.); rocioserrats@gmail.com (R.S.-L.); nuriamu@hotmail.com (N.M.-R.); affpezque@gmail.com (A.F.-F.); 2 Servicio de Cardiología, Hospital Politécnico Universitario La Fe, 46026 Valencia, Spain; serrano_ali@gva.es; 3 Centro de Atención Primaria de Burjassot, 46100 Valencia, Spain; minaya_alv@gva.es (A.S.M.-Z.); ramirez_sofcan@gva.es (S.R.-C.); 4 Servicio de Medicina Interna, Hospital Arnau de Vilanova, 46015 Valencia, Spain; herrera_ala@gva.es (A.H.-V.); llanos_alegar@gva.es (A.L.-G.); 5 Centro de Atención Primaria de Valterna (Paterna), 46980 Valencia, Spain; a.ariasfresneda@gmail.com

**Keywords:** cardiac rehabilitation, telemedicine, telerehabilitation, acute coronary syndrome, patient satisfaction, secondary prevention

## Abstract

**Background**: Extended telemonitored follow-up after acute coronary syndrome (ACS) has been shown to optimize secondary prevention outcomes. However, its impact on patient experience and outpatient visits remains unclear. **Methods**: This observational, retrospective, longitudinal study included 75 consecutive patients who underwent 10-month telemonitored follow-up after ACS and 50 consecutive patients who received standard care. Lipid parameters at hospital admission and 12 months post-discharge, patient experience (measured using the IEXPAC scale), and outpatient visits were evaluated. **Results**: The mean patient age was 58.0 years in the telemonitored group and 60.8 years in the control group, with males comprising 87% and 92%, respectively. The telemonitored group showed significant decreases in triglyceride levels (*p* < 0.011), VLDL cholesterol (*p* = 0.003), triglyceride/HDL ratio (*p* = 0.007), and remnant cholesterol levels (*p* = 0.018). The IEXPAC score was significantly higher in the telemonitored group (7.9 ± 1.5) compared to the standard care group (6.0 ± 1.9, *p* < 0.001). Higher ratings were observed across all domains: patient-professional productive interaction, the new patient-healthcare system relational model, and self-care. The telemonitored group also had fewer visits to Cardiology (1.0 ± 1.2 vs. 1.7 ± 1.0; *p* < 0.001) and Primary Care (7.1 ± 4.6 vs. 9.4 ± 5.2; *p* = 0.014). **Conclusions**: Extended telemonitored follow-up after ACS significantly enhances patient experience, improves lipid-related cardiovascular risk, and reduces outpatient visits to Primary Care and Cardiology compared to standard follow-up. These findings support the broader implementation of this healthcare pathway.

## 1. Introduction

International guidelines consistently underscore the primacy of non-pharmacological interventions—such as dietary changes, physical activity, smoking cessation, and psychosocial support—as first-line approaches following an acute coronary syndrome (ACS) [1]. Nevertheless, despite these strong recommendations, actual implementation and sustained adherence to such interventions remain inadequate in many clinical settings [2].

Multiple strategies have been explored to enhance follow-up care and improve long-term outcomes in patients with Chronic Coronary Syndrome (CCS). However, a significant challenge persists in maintaining consistent patient engagement and adherence over extended periods [3]. It is widely acknowledged that active patient involvement in self-management plays a pivotal role in improving cardiovascular outcomes [4]. This has brought increased attention to the role of structured cardiac rehabilitation (CR) programs.

Centre-Based Cardiac Rehabilitation (CBCR) programs are widely endorsed, with Class IA recommendations based on robust evidence demonstrating their efficacy in reducing cardiovascular morbidity and mortality [5]. Despite this, CBCR programmes are underutilised across various populations. Barriers contributing to this underuse include limited referral practices, geographic and logistical constraints, cost considerations, early return to occupational responsibilities, and persistent gender disparities in access and participation [6].

The emergence of the COVID-19 pandemic served as a significant catalyst for the rapid adoption of telemedicine across medical disciplines, including cardiac rehabilitation. In this context, the importance of home-based cardiac rehabilitation (HBCR) programs has become increasingly evident [7]. These programs now encompass a broad spectrum of modalities, from conventional in-hospital sessions to real-time remote consultations and asynchronous digital interactions facilitated by mobile health technologies.

More recently, hybrid models of cardiac rehabilitation have garnered interest. These combine in-person visits with virtual and remote sessions to create a flexible, patient-tailored care pathway [8]. While comparative studies have begun to assess the relative efficacy, adherence, and usability of these models, there remains a paucity of data specifically examining the patient experience—a critical factor for ensuring long-term success and healthcare improvement [9].

Improving the quality of care and achieving better health outcomes are fundamental objectives in modern healthcare systems. These goals are closely aligned with the principles of patient-centred care, which emphasize the integration of patients’ preferences, needs, and values into clinical decision-making [10,11]. Managing chronic conditions such as coronary heart disease is particularly challenging in the current healthcare landscape, given the increasing prevalence, rising healthcare resource consumption, and evolving patient expectations for personalised and accessible care [12,13].

Effective management strategies must ensure care continuity across various components of the healthcare system, from hospital to community and home-based settings [14]. In anticipation of the growing need for telemedicine, and prior to the COVID-19 pandemic, our team developed Cardioplan (Trilema Salud, Valencia, Spain)—a comprehensive telemonitoring solution designed specifically for community hospital cardiac rehabilitation units. The platform consists of a professional-facing web interface and a mobile application for patient use. Its aim is to facilitate remote engagement and sustained follow-up.

A validation study demonstrated superior outcomes with a 10-month telemonitored follow-up (TF) compared to standard follow-up (SF), typically involving a two-month hospital rehabilitation programme [15]. These findings supported the subsequent integration of the TF strategy into routine clinical practice. As part of an ongoing evaluation, the current objective is to assess the quality of this care model across three core dimensions: patient-perceived value of the intervention, which is essential for shaping patient-centred service improvements; clinical effectiveness, measured by changes in lipid profile parameters; and utilisation of healthcare resources, quantified through the frequency of outpatient visits.

## 2. Materials and Methods

### 2.1. Objectives

The primary objective of this study was to demonstrate the effectiveness of a novel care pathway based on extended TF in patients recovering from acute coronary syndrome (ACS), as compared to a SF model. The investigation focused on three principal outcomes: patient-reported experience and satisfaction with care delivery, improvement in cardiovascular risk as determined by lipid profile changes, and healthcare resource utilisation, specifically measured through outpatient consultations in cardiology and primary care settings during the post-discharge period. The hypothesis underlying this study posits that the extended TF model provides superior outcomes across all three domains relative to the conventional SF approach.

### 2.2. Design

This study employed a retrospective, longitudinal observational design involving two distinct patient cohorts treated at a single healthcare institution (Hospital Arnau de Vilanova, Valencia, Spain). The intervention group consisted of consecutive ACS patients who underwent the extended TF programme over a 10-month period. In contrast, the control group included the last consecutive patients managed under the SF pathway immediately before the implementation of the TF model, thereby ensuring temporal proximity and comparability between groups.

Data collection was conducted from September 2023 through June 2024. Patient-reported experience was assessed via telephone-administered surveys using the validated IEXPAC (Instrument for Evaluating the Experience of Chronic Patients) questionnaire [16]. Participants were asked to reflect on their care during the 12 months following hospital discharge. Clinical outcomes, particularly lipid parameters, were obtained from electronic medical records at hospital admission and again at 12 months post-discharge. Healthcare utilisation was evaluated through documented outpatient visits related to the index coronary event. This study followed the STROBE (Strengthening the Reporting of Observational Studies in Epidemiology) guidelines [17]. (Non-published Material).

### 2.3. Study Population

Participants were selected based on the following inclusion criteria: a confirmed diagnosis of ACS—specifically non-ST-elevation myocardial infarction (NSTEMI), ST-elevation myocardial infarction (STEMI), or unstable angina; evidence of coronary artery disease confirmed by diagnostic testing or intervention; and classification as low to moderate cardiovascular risk. Additional eligibility criteria included age ≤ 72 years with an ejection fraction (EF) ≥ 40%, smartphone with internet access and minimal technological proficiency. Exclusion criteria were refusal to give informed consent via telephone, heart failure, acute infectious diseases, uncontrolled supraventricular arrhythmias or ventricular tachycardias, and psychiatric or neurological disorders.

The TF cohort comprised 75 consecutive patients who completed the 10-month telemonitored follow-up protocol. Importantly, these patients were required to have concluded the follow-up programme at least two months prior to study enrolment to allow for a complete outcome assessment. The SF group consisted of 50 patients who had been discharged from hospital in the months immediately preceding the TF programme implementation, to ensure comparability. Patients in both cohorts were included if they had lipid levels measured between 10 and 12 months after hospital discharge, had provided informed consent, and had successfully completed the IEXPAC questionnaire.

The TF model was rapidly adopted upon its introduction, with over 85% of eligible patients enrolling in the programme. Given this widespread uptake, it was not feasible to recruit a concurrent control group for ethical and logistical reasons. Therefore, the SF cohort was retrospectively collected. Lipid profiles were taken from electronic health records between 10 and 12 months after hospital discharge, thus assuming this follow-up period.

### 2.4. Clinical Data Collection and Definitions

Comprehensive baseline data were extracted from hospital records and included sociodemographic characteristics, comorbidities, and cardiovascular risk factors. Smoking status was categorised as either “current smoker” or “former smoker,” the latter defined as individuals who had quit smoking within the past three years. Diabetes mellitus, hypertension, and hypercholesterolaemia were defined according to prevailing clinical diagnostic criteria.

Cardiac function was evaluated through echocardiographic assessments. In instances where the echocardiographic report described systolic function as “normal,” a standardised EF of 60% was assigned for consistency in data analysis.

### 2.5. Assessment of Lipid Parameters

To evaluate the clinical effectiveness of the TF programme, serial measurements of lipid parameters were obtained from both groups. The parameters assessed included total cholesterol, high-density lipoprotein cholesterol (HDL-C), low-density lipoprotein cholesterol (LDL-C), very-low-density lipoprotein cholesterol (VLDL-C), and triglycerides. In addition to individual lipid values, calculated ratios were also analysed: total cholesterol to HDL-C, triglycerides to HDL-C, and remnant cholesterol levels. All laboratory analyses were conducted in accordance with standardised hospital operating procedures and quality control protocols.

### 2.6. Patient Experience Evaluation

Patient-reported experience was systematically assessed using the IEXPAC 2018 (11 + 4) questionnaire [16]. This validated tool captures the quality of patient–provider interactions, the integration of digital health tools, and the promotion of self-care. Specifically, questions 1, 2, 5, and 9 assessed relational quality with healthcare professionals; questions 3, 7, and 11 evaluated the digital health model; and questions 4, 6, 8, and 10 pertained to self-management behaviours. Questions 12 and 13 addressed follow-up via telephone post-discharge and were scored separately. Items 14 and 15 were excluded from analysis, as they referred to home-based treatments not applicable to this study population. Surveys were conducted through telephone interviews to maximise response rates and ensure data completeness. The TF group was contacted between 12 and 20 months after hospital discharge, while the SF group was interviewed between 18 and 29 months post-discharge. Online and postal questionnaire attempts yielded response rates of only 40% and 52%, respectively [18], prompting a shift to telephone-based data collection. This change was made to address the limited number of eligible patients and reduce non-responses, lack of cooperation, and incomplete or inaccurate answers [19]. To minimise potential bias, half of the interviews in each group were conducted by cardiologists and half by primary care physicians, none of whom had prior contact with the patient. All patients were informed about this study’s objectives, assured of anonymity, and provided verbal consent prior to participation. Responses were directly entered into the IEMAC-IEXPAC online platform. For further details on the IEXPAC scale, see the Supplementary File S1.

### 2.7. Measurement of Outpatient Resource Utilisation

Healthcare resource utilization was quantified by counting outpatient visits to both Primary Care and Cardiology clinics. Only visits explicitly related to the index coronary event were included. Both in-person and telephone consultations were considered. The inclusion timeframe covered the first 12 months following hospital discharge. Data were retrieved from the institution’s electronic health record system to ensure completeness and accuracy.

### 2.8. Description of the Telemonitored Follow-Up Programme

The TF programme involved a combination of in-person assessments and prolonged remote monitoring via a custom-designed mobile health application. The programme began with a baseline hospital visit, during which patients underwent stress testing, comprehensive blood analysis, anthropometric measurements, and were introduced to the functionalities of the mobile application.

A second in-person group visit was conducted, during which patients participated in structured sessions covering secondary prevention strategies, reinforcing patient education, and engaging in aerobic and resistance training. The aerobic protocol required patients to walk along an 80-m corridor while maintaining 55–65% of their heart rate reserve, calculated using the Karvonen formula. Patients wore chest sensors that transmitted real-time data to the app’s exercise module.

Ongoing telemonitoring continued over a 10-month period. Patients used the app to report data on blood pressure, heart rate, weight, waist circumference, medication adherence, smoking status, dietary habits, and psychological well-being. The app also included a secure messaging function for text and video communication with healthcare providers, as well as educational materials and links to relevant websites. A scheduled blood test at three months allowed for remote optimisation of lipid-lowering therapies. Final evaluations were conducted at the end of the programme. All data were encrypted and securely stored on hospital servers.

The SF group received routine discharge instructions in line with current secondary prevention guidelines [5] and attended follow-up appointments in Primary Care and Cardiology clinics based on standard scheduling practices. No structured remote monitoring or digital health interventions were employed.

### 2.9. Statistical Analysis

The minimum recommended sample size for each study group was set at 50 patients, in alignment with the guidelines established by the developers of the IEXPAC scale. This sample size permits estimation of mean IEXPAC scores with a 95% confidence interval and a margin of error of approximately 0.5 units, assuming a standard deviation of 1.8. The TF group sample was increased to 75 patients to enhance the precision of results and reduce the margin of error to 0.41 units. The sample size was determined according to the IEXPAC scale and was not statistically powered for comparisons of lipid parameters. Statistical analyses were conducted using R software, version 4.4.1 (R Foundation for Statistical Computing, Vienna, Austria). There were no missing data points for the lipid variables. Descriptive statistics were used to summarise all study variables. Continuous variables are presented as means ± standard deviations or medians with interquartile ranges, depending on data distribution. Categorical variables are expressed as absolute counts and percentages.

Group comparisons were conducted using independent samples *t*-tests (Welch’s *t*-test in cases where the assumption of equal variances could not be met), and Fisher’s exact test. To examine the independent effect of follow-up model (TF vs. SF) on lipid outcomes, multivariate linear regression models were developed, adjusting for potential confounders such as ezetimibe therapy. All statistical tests were two-sided, and a *p*-value < 0.05 was considered indicative of statistical significance.

## 3. Results

### 3.1. Study Cohorts

To include 75 consecutive patients from the 10-month TF programme and 50 SF patients after hospital discharge at Hospital Arnau de Vilanova, a total of 139 telephone calls were placed to potential study participants. Out of the 139 individuals contacted, 9 did not respond to the calls, and of the remaining 130 patients, 125 consented to participate and completed the questionnaire, resulting in a high response rate of 96%. Only 5 patients declined to be part of this study after initial contact.

**Figure 1:**
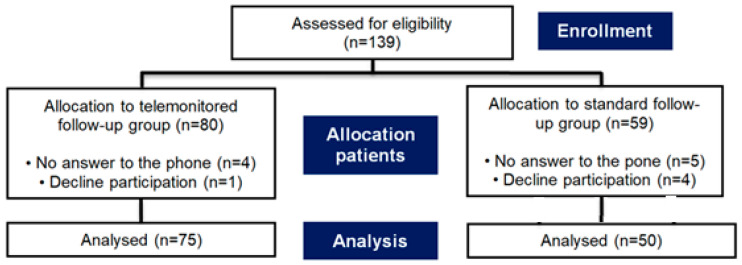
CONSORT diagram showing the flow of participants.

The enrolment process and subsequent flow of participants are illustrated in the CONSORT diagram provided in Figure 1.

### 3.2. Baseline Characteristics

The mean age of patients was 58.0 ± 9.4 years in the telemonitored group and 60.8 ± 7.9 years in the control group, with males constituting 86.7% and 92.0% of the respective groups. Although the average age was lower in the TF group, the difference was not statistically significant (*p* = 0.079).

The prevalence of cardiovascular risk factors, such as hypertension, diabetes, smoking status, and dyslipidaemia, did not differ significantly between the groups. However, some risk factor data were incomplete due to the retrospective design of this study and missing entries in medical records, limiting full comparability in this domain.

Medication regimens at discharge included high-potency statins in most patients across both groups, with treatment being adjusted during follow-up to comply with current guideline-based targets for lipid management. Notably, a statistically significant difference (*p* = 0.007) was observed in the prescription of ezetimibe, which was more commonly used in the TF group (82.7%) compared to the SF group (60.0%). Despite this, the average number of medications per patient did not differ significantly between groups, with the TF group averaging 5.8 ± 2.3 medications and the SF group 5.4 ± 2.1 (*p* = 0.387). The detailed baseline clinical and therapeutic profiles are summarised in Table 1.

### 3.3. Lipid Parameters

To evaluate the clinical efficacy of cardiovascular prevention strategies in both groups, lipid profile values were considered. This study revealed that both the TF and SF groups successfully achieved acceptable levels of total cholesterol and LDL-C, with LDL-C decreasing by 61% from hospital admission in the SF group and by 48% in the TF group, in accordance with clinical guidelines. No significant differences were observed between the groups. Despite the similar outcomes in LDL-C and total cholesterol, the higher prevalence of ezetimibe use in the TF group was identified as a potential confounding factor and was taken into consideration during the analysis of lipid outcomes. Although HDL-C levels increased more in the TF group than in the SF group, this improvement did not reach statistical significance (*p* = 0.121).

Several lipid-related parameters improved significantly in the TF group. There was a marked reduction in triglyceride levels (*p* = 0.011) and VLDL-C (*p* = 0.003) compared to the SF group. Moreover, the triglyceride-to-HDL-C ratio, was significantly lower in the TF group (*p* = 0.007). The level of remnant cholesterol particles, recognized as contributors to residual cardiovascular risk, was also significantly lower in the TF group compared to the SF group (*p* = 0.018).

Other risk factors were not included in the comparison due to missing data in medical records, attributed to the retrospective nature of this study. These results are documented in Table 2.

### 3.4. Patient Experience

Patient-reported experience, assessed using the 13 individual questions of the IEXPAC scale, revealed that the TF group scored significantly higher on 11 of the 13 items, indicating a more favourable experience. The overall mean IEXPAC score was 7.9 ± 1.5 in the TF group, compared to 6.0 ± 1.9 in the SF group, *p* < 0.001.

**Figure 2:**
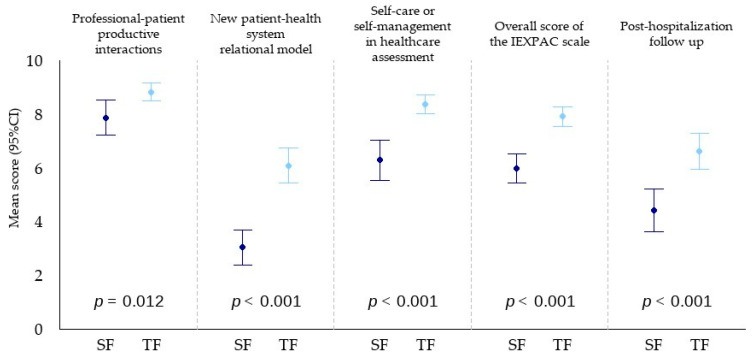
Analysis of patient experience. Plotting results of the IEXPAC scale. Mean (SD). From left to right: first graph, relational quality with healthcare professionals (questions 1, 2, 5, and 9); second graph, evaluation of the digital health model (questions 3, 7, and 11); third graph, assessment of self-management behaviours (questions 4, 6, 8, and 10); fourth graph, overall score. SF: standard follow-up (dark blue); TF: telemonitored follow-up (light blue).

When responses were categorised according to key assessment domains, the TF group demonstrated statistically superior outcomes in the productive patient-professional interactions (8.8 ± 1.4 vs. 7.9 ± 2.3, *p* = 0.012), the new patient-healthcare system relational model (6.1 ± 2.8 vs. 3.1 ± 2.3, *p* < 0.001), and self-care or health self-management (8.4 ± 1.5 vs. 6.3 ± 2.6, *p* < 0.001). (Figure 2). Scores for questions 12 and 13, which relate to the experience of immediate care after discharge, were also higher in the TF group: 6.6 ± 2.9, than in the SF group: 4.4 ± 2.8, (*p* < 0.001). For detailed IEXPAC scale scores for both patient groups, see Supplementary Table S1.

### 3.5. Outpatient Visits

**Figure 3:**
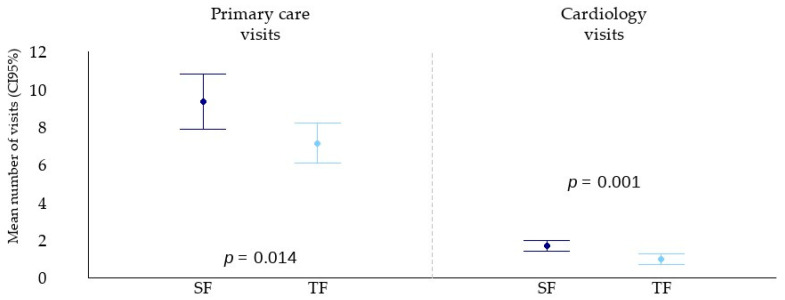
Analysis of outpatient visits. Plotting results of outpatient healthcare utilisation. Mean (SD). From left to right: first graph, Primary Care visits; second graph, Cardiology visits. SF: standard follow-up (dark blue); TF: telemonitored follow-up (light blue).

A significantly lower number of Primary Care visits was recorded in the TF group compared to the SF group (7.1 ± 4.6 vs. 9.4 ± 5.2; *p* = 0.014). Notably, there were substantial differences in the number of telephone consultations, likely reflecting differences in healthcare management practices: 1.2 ± 1.5 in the TF group vs. 2.6 ± 2.3 in the SF group (*p* < 0.001). In contrast, differences in the number of in-person consultations were less pronounced (5.9 ± 4.3 vs. 6.8 ± 4.3; *p* = 0.282), possibly due to the inclusion of visits for sick leave certificate renewals. Additionally, the TF group had a significantly lower number of in-person Cardiology visits (1.0 ± 1.2 vs. 1.7 ± 1.0; *p* < 0.001) (Figure 3).

## 4. Discussion

New hybrid models of CR, which integrate in-person sessions with telecare modalities, are progressively transforming the landscape of secondary prevention strategies, replacing traditional hospital-based programmes [20]. While both hospital-based and telemonitoring-based rehabilitation programmes have demonstrated comparable clinical effectiveness [21], home-based interventions offer distinct advantages. These include greater accessibility, reduced travel burden, improved adherence, and a potential reduction in hospital readmissions [22]. Despite a growing body of evidence confirming the non-inferiority of HBCR, telehealth programmes leveraging mobile health technologies remain classified as Class IIb recommendations in the most recent European Society of Cardiology (ESC) Guidelines on cardiovascular disease prevention [5].

A significant achievement of TF programmes is their capacity to enrol a higher proportion of low- to moderate-risk patients. This contrasts sharply with traditional CBCR, where enrolment remains limited. The EUROSPIRE V registry, for instance, reported participation rates of merely 34% among eligible patients [23]. The TF model implemented in this study achieved enrolment rates exceeding 85% in this patient group, demonstrating the feasibility and scalability of this approach in real-world clinical settings.

Traditional CBCR programmes are generally limited to two- to three-month durations, although some may extend up to six months. In contrast, our study examined a 10-month TF strategy, which provided patients with a sustained, structured programme of remote monitoring, educational support, and healthcare provider interaction. Evidence suggests that such long-term engagement may yield durable improvements in health behaviours and clinical outcomes. Cruz-Cobo et al. found that a six-month mHealth intervention significantly improved lifestyle habits and selected clinical parameters compared to SF after ACS [24]. Moreover, previous research highlights that benefits from short-term interventions tend to wane over time, reinforcing the necessity of extended post-discharge care [25]. A meta-analysis by Zhong et al. showed that telemonitoring CR programmes lasting up to six months improved functional capacity albeit with no significant differences in the control of cardiovascular risk factors [26].

There is also a well-documented dose-response relationship in CBCR, wherein increased participation is associated with improved outcomes [27].

This principle may logically extend to digital interventions. The prolonged telemonitoring strategy can be considered not merely as an extension of conventional cardiac rehabilitation, or even a hybrid model, but as a distinct post-acute care pathway following an ACS. This model represents a shift toward integrated, patient-centered care that emphasizes continuous clinical oversight and proactive risk management beyond hospital discharge. A prospective, single-centre clinical trial is ongoing within our Health Department to investigate the feasibility, safety, and clinical impact of implementing this telemonitoring strategy to enhance continuity of care following hospital discharge (NCT05875311).

Our study extends these findings by demonstrating significant changes in non-traditional lipid markers—namely, the triglyceride-to-HDL-C ratio and remnant cholesterol levels—which are increasingly recognised as strong predictors of cardiovascular events and mortality [28,29,30,31].

Notably, to our knowledge, this is the first study to report improvements in these lipid ratios as a direct outcome of a digital CR intervention. These favourable changes occurred independently of ezetimibe use [32], suggesting that the TF programme itself contributed to better lipid control. These results build upon prior findings from our group, in which extended TF reduced not only triglyceride/HDL-C ratios but also GlycA levels, an emerging inflammatory biomarker associated with cardiovascular and all-cause mortality [33,34]. In the context of widespread high-potency statin use, additional lipid targets such as triglyceride-rich lipoproteins and inflammatory markers become particularly relevant. This underscores the potential of telemonitoring to complement pharmacologic therapies in addressing residual cardiovascular risk.

These findings are in line with recent Class IIa recommendations supporting the use of mobile health interventions to facilitate long-term adherence to cardiovascular prevention measures and reduce hospital readmissions [35]. The extended TF model in this study likely contributes to these outcomes by fostering continuous patient engagement, regular health data tracking, timely feedback from clinicians, and reinforcement of lifestyle modifications.

Beyond clinical outcomes, patient-reported experience is increasingly recognised as a vital metric for evaluating healthcare quality. In this regard, the TF group exhibited a significantly more favourable experience compared to the SF group, as assessed using the validated IEXPAC scale, with improvements across three key healthcare aspects: productive patient-professional interactions, the digital health relational model, and self-management post-hospital discharge, all reflecting a greater perception of support.

Importantly, this is the first study to assess patient experience following post-ACS telemonitoring using a standardised metric. The IEXPAC scale evaluates the quality of care received from healthcare providers and the organisation of care, both in hospital and primary care settings. The tool was selected due to its relevance to post-discharge patients and its applicability to evolving patient-healthcare system interactions, particularly in the context of digital health and chronic disease management. The IEXPAC score highlights the role of frequent patient-provider interactions and educational resources in promoting self-care behaviours, independent of the direct clinical effectiveness of TF.

There are demographic factors that may influence patient experience, such as age and sex. In our study, non-significant differences between groups did not appear to contribute to the variation in IEXPAC scores [36]. Other potential determinants not assessed in this study include marital status and educational level [37,38]. Both adult cohorts were recruited from the same peri-urban region and neighbouring towns, ensuring comparability in these characteristics.

Contrary to initial assumptions, the improved patient experience reported in the TF group was associated with fewer in-person visits to Cardiology and Primary Care. This contradicts the assumption that more frequent contact is necessary for higher satisfaction and reinforces the potential for digital health tools to optimise care delivery while reducing system burden [39].

Given the growing strain on primary care resources and the rising prevalence of chronic conditions, scalable telehealth solutions may offer a cost-effective mechanism for supporting patients outside traditional clinical environments. In this context, the results of our economic evaluation demonstrate that Cardioplan system leads to cost savings from both the perspective of the Spanish National Health System and the societal perspective, with comparable effectiveness. Therefore, it represents a dominant option from both viewpoints when compared to center-based cardiac rehabilitation [40].

This study supports calls from global health organisations and clinical societies to develop sustainable, patient-centred solutions that extend beyond episodic care. Integrating mobile applications with structured professional oversight enables frequent, low-friction interactions that reinforce behaviour change and promote long-term adherence to secondary prevention goals. Our results demonstrate the feasibility and effectiveness of such an approach in real-world clinical practice.

## 5. Conclusions

This investigation demonstrates that a structured, 10-month TF strategy—comprising just two hospital-based sessions—confers superior benefits in terms of patient experience, lipid risk profile improvement, and reduced outpatient service utilisation compared to SF after ACS. These findings highlight the potential of digital health interventions to enhance both the quality and efficiency of post-ACS care, providing a model for wider adoption within modern healthcare systems.

## 6. Limitations

While the findings are promising, several limitations must be acknowledged. First, the retrospective and observational nature of this study, using existing data, may introduce potential selection bias. Although all eligible patients in the TF cohort were consecutively included, the SF group was formed from consecutive patients discharged prior to TF implementation, and residual confounding cannot be excluded. Second, the relatively small sample size—especially in the control group—may limit the generalisability of the results. Third, the time between discharge and questionnaire administration varied between groups, potentially influencing recall bias in patient experience ratings. Finally, the analysis did not adjust for potential effects of comorbidities or socioeconomic factors, which may have influenced patient-reported outcomes.

Nevertheless, the consistency of our findings across multiple outcome domains underscores the potential of extended telemonitoring to deliver high-value care in post-ACS populations. Future prospective, multicentre studies with larger cohorts are warranted to confirm these results and further refine implementation strategies for telehealth-based cardiac rehabilitation programmes.

## Figures and Tables

**Table 1:** Baseline patient characteristics.

Variable		Standard Follow-Up	Telemonitored Follow-Up	*p*-Value
Patients [*n* (%)]		50 (100.0%)	75 (100.0%)	
Sex [*n* (%)]	Male	46 (92.0%)	65 (86.7%)	0.402
	Female	4 (8.0%)	10 (13.3%)	
Age (years)	Mean (SD)	60.8 (7.9)	58.0 (9.4)	0.079
	Median (Range)	61 (41–75)	58 (34–75)	
Body Mass Index	Mean (SD)	28.1 (4.0)	27.9 (4.6)	0.864
	Median (Range)	28 (21–39)	27 (20–43)	
Hypertension [*n* (%)]	No	24 (48.0%)	36 (48.0%)	1.000
	Yes	26 (52.0%)	39 (52.0%)	
Diabetes Mellitus [*n* (%)]	No	40 (80.0%)	57 (76.0%)	0.666
	Yes	10 (20.0%)	18 (24.0%)	
Dyslipidemia [*n* (%)]	No	25 (50.0%)	38 (50.7%)	1.000
	Yes	25 (50.0%)	37 (49.3%)	
Smoking [*n* (%)]	No	24 (48.0%)	37 (49.3%)	1.000
	Yes	26 (52.0%)	38 (50.7%)	
AMI [*n* (%)]	No	11 (22.0%)	17 (22.7%)	1.000
	Yes	39 (78.0%)	58 (77.3%)	
UA [*n* (%)]	No	40 (80.0%)	58 (77.3%)	0.826
	Yes	10 (20.0%)	17 (22.7%)	
Statin treatment [*n* (%)]	None	6 (12.0%)	2 (2.7%)	0.120
	Atorvastatin 80 mg	17 (34.0%)	23 (30.7%)	
	Atorvastatin 40 mg	7 (14.0%)	19 (25.3%)	
	Rosuvastatin 20 mg	20 (40.0%)	31 (41.3%)	
Ezetimibe treatment [*n* (%)]	No	20 (40.0%)	13 (17.3%)	0.007
	Yes	30 (60.0%)	62 (82.7%)	
LVEF (%)	Mean (SD)	57.1 (7.7)	57.3 (6.6)	0.912
	Median (Range)	60 (35–70)	60 (40–75)	

AMI = acute myocardial infarction; UA = unstable angina; LVEF = left ventricular ejection fraction; SD = standard deviation.

**Table 2:** Analysis of lipid parameters.

Lipid Parameter	Time Reference	Standard Follow-Up	Telemontored Follow-Up	*p*-Value	*p*-Value *
Total cholesterol (mg/dL) [mean (SD)]	Hospital admission	179.4 (47.0)	166.2 (43.3)	0.109	0.072
	12-month post-discharge	138.6 (52.3)	125.8 (30.0)	0.087	0.096
	Decrease during follow-up	40.9 (57.4)	40.4 (48.5)	0.958	0.800
HDL cholesterol (mg/dL) [mean (SD)]	Hospital admission	44.9 (12.6)	41.1 (10.6)	0.071	0.065
	12-month post-discharge	47.4 (10.0)	46.3 (11.5)	0.578	0.526
	Decrease during follow-up	−2.54 (10.1)	−5.23 (8.6)	0.112	0.121
LDL cholesterol (mg/dL) [mean (SD)]	Hospital admission	106.4 (44.5)	95.6 (38.8)	0.155	0.093
	12-month post-discharge	65.0 (44.9)	57.9 (21.9)	0.242	0.212
	Decrease during follow-up	41.4 (54.6)	37.7 (41.3)	0.670	0.544
VLDL cholesterol (mg/dL) [mean (SD)]	Hospital admission	27.3 (10.6)	29.2 (12.0)	0.371	0.311
	12-month post-discharge	25.9 (13.1)	21.1 (11.5)	0.034	0.051
	Decrease during follow-up	1.41 (10.2)	8.11 (12.6)	0.002	0.003
Remnant cholesterol (mg/dL) [mean (SD)]	Hospital admission	28.1 (11.6)	29.5 (12.0)	0.536	0.415
	12-month post-discharge	26.1 (13.7)	21.7 (12.9)	0.070	0.140
	Decrease during follow-up	2.00 (10.6)	7.76 (13.2)	0.011	0.018
Triglycerides (mg/dL) [mean (SD)]	Hospital admission	141.0 (57.6)	143.7 (53.3)	0.787	0.788
	12-month post-discharge	140.4 (81.0)	105.1 (52.9)	0.004	0.011
	Decrease during follow-up	0.6 (58.0)	38.6 (50.9)	<0.001	0.001
Total cholesterol/HDL ratio [mean (SD)]	Hospital admission	4.30 (1.73)	4.23 (1.36)	0.826	0.719
	12-month post-discharge	2.99 (1.04)	2.84 (0.95)	0.412	0.510
	Decrease during follow-up	1.31 (1.75)	1.39 (1.34)	0.752	0.944
Triglycerides/HDL ratio [mean (SD)]	Hospital admission	3.49 (1.90)	3.77 (1.74)	0.384	0.397
	12-month post-discharge	3.17 (2.30)	2.48 (1.58)	0.051	0.100
	Decrease during follow-up	0.32 (1.90)	1.29 (1.59)	0.002	0.007

HDL = high-density lipoprotein; LDL = low-density lipoprotein; VLDL = very-low-density lipoprotein; SD = standard deviation; *p*-Value *: controlled by ezetimibe treatment.

## Data Availability

All data supporting the findings of this study are available within this article and its Supplementary Materials. Some raw data are available from the corresponding author upon reasonable request; however, certain datasets are part of an ongoing study and are not publicly available currently.

## Supplementary Materials

The following supporting information can be downloaded at: https://www.mdpi.com/article/10.3390/jcm14207283/s1, File S1: Document iexpac-english.pdf; Table S1: Analysis of patient experience.
