# Supplementary material for: Extended Telemonitored Follow-Up After Acute Coronary Syndrome: A Healthcare Pathway That Improves Cardiovascular Prevention and Patient Experience, and Reduces Outpatient Visits
Source: J Clin Med. 2025 Oct 15;14(20):7283. doi: 10.3390/jcm14207283 (PMC12565088; doi:10.3390/jcm14207283)
Supplement: Supplementary file 1 [file jcm-14-07283-s001.zip › jcm-3905249-supplementary File S1.pdf]

# SCALE IEXPAC

## ESCALA IEXPAC

Chronic Patient Experience Evaluation Instrument

IEXPAC ©2015

Institut Català de la Salut

MSD

b+oef berrikuntza + ikerketa + osasuna eusko fundazioa fundación vasca de innovación e investigación sanitarias

UNIVERSITAS Miguel Hernández

## INSTRUCTIONS

To respond to the statements, please read each one in the order in which they appear and choose the option that best reflects your opinion. There are no right or wrong answers, it is your own personal experience that matters. All statements refer to the last 6 months.

The statements refer at all times to the group of professionals who care for you. To answer, think about your doctor and nurse at the health centre, the specialists and nurses at the hospital, pharmacists, the case manager, physiotherapist, psychologist; think also about other professionals such as the social worker at the health centre or at the municipality social services and, generally, about the whole group of people who care for you. Think about these people whenever we mention the professionals who care for you.

Your answers will be kept confidential and will not be shared with the professionals who care for you.

Mark with an X the option that best reflects your personal opinion. Please do not leave any statements unanswered.

**Thank you very much for your cooperation.**

## STATEMENTS

| **Based on your experience as a chronic patient, please respond to the following statements by marking the frequency that each situation applies to you.** | Never | Almost never | Sometimes | Almost always | Always |
|---|---|---|---|---|---|
| **1. They respect my lifestyle**<br>The professionals who care for me listen to me and ask me about my needs, habits and preferences to adapt my treatment and care plan. | ○ | ○ | ○ | ○ | ○ |
| **2. They are coordinated to offer me good care**<br>The professionals who care for me at the health centre and those who care for me at the hospital talk to each other and coordinate to improve my wellbeing and quality of life. | ○ | ○ | ○ | ○ | ○ |
| **3. They help me become informed via the Internet**<br>The professionals who care for me inform me about reliable websites and Internet forums that I can consult to better understand my disease, its treatment and the consequences they may have on my life. | ○ | ○ | ○ | ○ | ○ |
| **4. I now know how to look after myself better**<br>With the support of my professionals, I feel now that I have more confidence in my ability to take care of myself, manage my health problems and keep my autonomy. | ○ | ○ | ○ | ○ | ○ |
| **5. They ask me about and help me follow my treatment plan**<br>I review the adherence to my treatment and care plan with the professionals who care for me, and if I have questions, they answer them. | ○ | ○ | ○ | ○ | ○ |
| **6. We agree on objectives to lead a healthy life and to control my health problems better**<br>I've been able to agree with the professionals who care for me on specific objectives regarding diet, physical exercise and medication to control my health problems better. | ○ | ○ | ○ | ○ | ○ |
| **7. I use the Internet and my mobile phone to consult my clinical record**<br>I use the Internet and my mobile phone to consult my clinical record, test results, scheduled visits and to access other services on my health service's website | ○ | ○ | ○ | ○ | ○ |
| **8. They ensure that I take my medication correctly**<br>The professionals who care for me review with me all the medications I take, how I take them, how they make me feel, and I can ask them about the questions I have. | ○ | ○ | ○ | ○ | ○ |
| **9. They are concerned about my wellbeing**<br>The professionals who care for me are concerned with my quality of life and I feel they are committed to improving my wellbeing. | ○ | ○ | ○ | ○ | ○ |
| **10. They inform me about health and social resources that can help me**<br>The professionals who care for me inform me about the health and social resources available (in my neighbourhood, town or city) that I can use to improve my health problems and take better care of myself. | ○ | ○ | ○ | ○ | ○ |
| **11. They encourage me to talk with other patients**<br>The professionals who care for me encourage me to participate in patients groups to share information and experiences on how to care for ourselves and improve our health. | ○ | ○ | ○ | ○ | ○ |

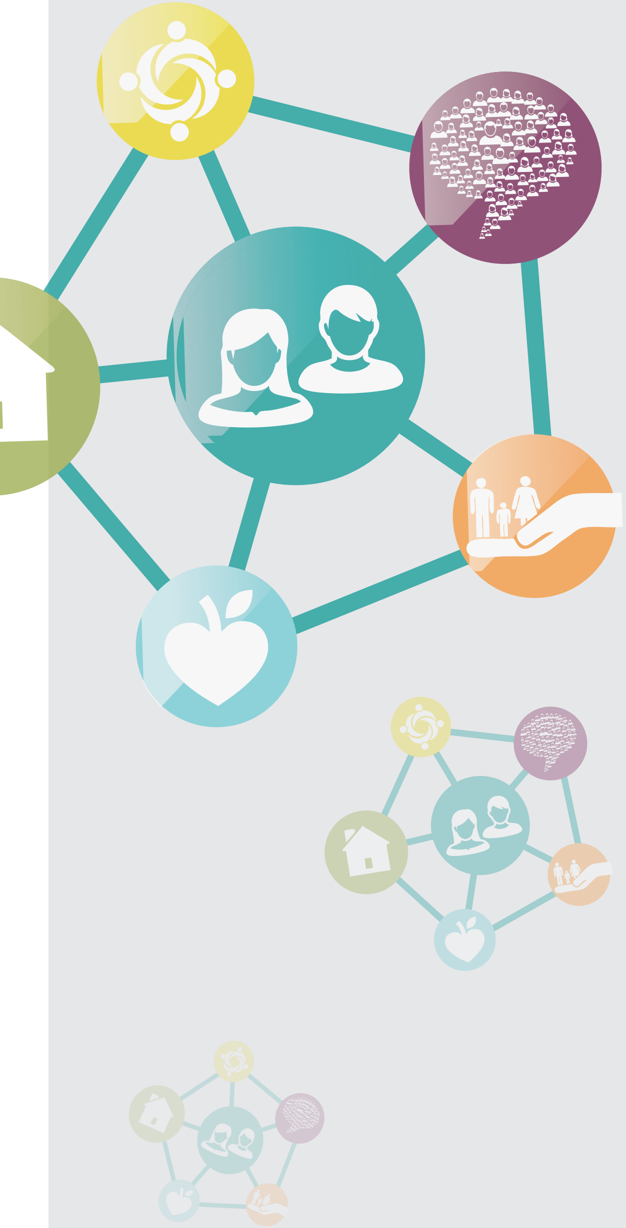

## STATEMENTS

**Based on your experience as a chronic patient, please respond to the following statements by marking the frequency that each situation applies to you.**

| | Never | Almost never | Sometimes | Almost always | Always |
|---|---|---|---|---|---|
| If you have been hospitalized in the last 6 months, please respond to the following statement:<br>**12. They care about me upon my arrival home after being in hospital**<br>After being discharged from hospital, they have called or visited me at home to see how I was and what care I needed. | ○ | ○ | ○ | ○ | ○ |
| If you have received emergency care in the last 6 months, please respond to the following statement:<br>**13. They counsel me on how to avoid a new emergency**<br>After having been in the emergency room, the professionals who care for me know what has happened to me and advise me on how to avoid a new emergency. | ○ | ○ | ○ | ○ | ○ |
| If you have received healthcare in your home in the last 6 months, please respond to the following statement:<br>**14. They care for me well in my home**<br>The professionals who care for me in my home try to solve my health problems in coordination with the professionals of the health centre and the hospital. | ○ | ○ | ○ | ○ | ○ |
| If you have received care of the social services in the last 6 months, please respond to the following statement:<br>**15. Social services are coordinated with the health services to provide me with good care**<br>The professionals who care for me in social services talk to and coordinate with the healthcare professionals to provide me with good care. | ○ | ○ | ○ | ○ | ○ |

**Sex:** ______ **Age:** ______ **Number of medications you are taking:** ______
