# Supplementary material for: Extended Telemonitored Follow-Up After Acute Coronary Syndrome: A Healthcare Pathway That Improves Cardiovascular Prevention and Patient Experience, and Reduces Outpatient Visits
Source: J Clin Med. 2025 Oct 15;14(20):7283. doi: 10.3390/jcm14207283 (PMC12565088; doi:10.3390/jcm14207283)
Supplement: Supplementary file 1 [file jcm-14-07283-s001.zip › jcm-3905249-supplementary Table S1.pdf]

Supplementary Table S1. Analysis of patient experience

		Standard follow-up	Telemonitored follow-up	*p*-Value
C1. They respect my lifestyle	Mean (SD)	8.4 (2.7)	9.1 (1.5)	0.116
	Median (Range)	10 (0-10)	10 (5-10)	
C2. They are coordinated to offer me good care	Mean (SD)	7.4 (3.1)	7.9 (3.1)	0.382
	Median (Range)	10 (0-10)	10 (0-10)	
C3. They help me become informed via the Internet	Mean (SD)	2.9 (3.9)	5.4 (4.2)	**0.001**
	Median (Range)	0 (0-10)	5 (0-10)	
C4. I now know how to look after myself better	Mean (SD)	8.0 (2.9)	9.2 (1.7)	**0.008**
	Median (Range)	10 (0-10)	10 (0-10)	
C5. They ask me about and help me follow my treatment plan	Mean (SD)	7.8 (3.3)	9.0 (2.2)	**0.019**
	Median (Range)	10 (0-10)	10 (0-10)	
C6. We agree on objectives to lead a healthy life and to control my health problems better	Mean (SD)	6.6 (3.8)	9.6 (1.3)	**<0.001**
	Median (Range)	7.5 (0-10)	10 (2.5-10)	
C7. I use the Internet and my mobile phone to consult my clinical record	Mean (SD)	4.9 (4.3)	7.5 (3.5)	**0.001**
	Median (Range)	5 (0-10)	10 (0-10)	
C8. They ensure that I take my medication correctly	Mean (SD)	7.0 (3.7)	9.0 (1.9)	**0.001**
	Median (Range)	10 (0-10)	10 (0-10)	
C9. They are concerned about my wellbeing	Mean (SD)	8.0 (3.1)	9.3 (1.7)	**0.006**
	Median (Range)	10 (0-10)	10 (2.5-10)	
C10. They inform me about health and social resources that can help me	Mean (SD)	3.6 (4.0)	5.7 (3.9)	**0.005**
	Median (Range)	2.5 (0-10)	5 (0-10)	
C11. They encourage me to talk with other patients	Mean (SD)	1.4 (3.0)	5.4 (3.8)	**<0.001**
	Median (Range)	0 (0-10)	5 (0-10)	
C12. They care about me upon my arrival home after being in hospital	Mean (SD)	2.5 (4.0)	4.7 (4.2)	**0.003**
	Median (Range)	0 (0-10)	5 (0-10)	
C13. They counsel me on how to avoid a new emergency	Mean (SD)	6.4 (4.0)	8.5 (2.7)	**0.002**
	Median (Range)	7.5 (0-10)	10 (0-10)	
Productive patient-professional interactions (C1, C2, C5, C9)	Mean (SD)	7.9 (2.3)	8.8 (1.4)	**0.012**
	Median (Range)	8.8 (0-10)	9.4 (3.8-10)	
New patient-healthcare system relational model (C3, C7, C11)	Mean (SD)	3.1 (2.3)	6.1 (2.8)	**<0.001**
	Median (Range)	3.3 (0-8.3)	6.7 (0-10)	
Self-care or health self-management (C4, C6, C8, C10)	Mean (SD)	6.3 (2.6)	8.4 (1.5)	**<0.001**
	Median (Range)	6.3 (0.6-10)	8.8 (3.1-10)	
Post-hospitalization follow-up (C12-C13)	Mean (SD)	4.4 (2.8)	6.6 (2.9)	**<0.001**
	Median (Range)	5.0 (0-10)	7.5 (0-10)	
Overall score on the IEXPAC scale (C1-C11)	Mean (SD)	6.0 (1.9)	7.9 (1.5)	**<0.001**
	Median (Range)	6.1 (0.9-9.6)	8.2 (2.7-10)	
